# Dietary patterns and severity of symptom with the risk of esophageal squamous cell carcinoma and its histological precursor lesions in China: a multicenter cross-sectional latent class analysis

**DOI:** 10.1186/s12885-022-09206-y

**Published:** 2022-01-21

**Authors:** Zhaoping Zang, Yong Liu, Jialin Wang, Yuqin Liu, Shaokai Zhang, Yongzhen Zhang, Liwei Zhang, Deli Zhao, Fugang Liu, Lina Chao, Xinzheng Wang, Chunli Zhang, Guohui Song, Zhiyi Zhang, Youpeng Li, Zheng Yan, Yongxiu Wen, Yinyin Ge, Chen Niu, Wei Feng, Rena Nakyeyune, Yi Shen, Yi Shao, Xiuhua Guo, Aiming Yang, Fen Liu, Guiqi Wang

**Affiliations:** 1 Department of Epidemiology and Health Statistics, School of Public Health, Beijing Municipal Key Laboratory of Clinical Epidemiology, Capital Medical University, Beijing, 100069 China; 2 Department of Endoscopy, National Cancer Center/National Clinical Research Center for Cancer/Cancer Hospital, Chinese Academy of Medical Sciences and Peking Union Medical College, Beijing, 100021 China; 3 Shandong Cancer Hospital and Institute, Shandong First Medical University and Shandong Academy of Medical Sciences, Shandong, 250000 China; 4 Gansu Provincial Cancer Hospital, Gansu, 730000 China; 5 Department of Cancer Epidemiology and Prevention, The Affiliated Cancer Hospital of Zhengzhou University, Henan Cancer Hospital, Henan, 450008 China; 6 Department of Epidemiology, Shanxi Cancer Hospital, Shanxi, 030013 China; 7 The Fourth Hospital of Hebei Medical University, Hebei, 050000 China; 8 Feicheng People’s Hospital, Shandong, 271600 China; 9 Dongping People’s Hospital, Shandong, 271500 China; 10 Department of Epidemiology, Hebi People’s Hospital, Henan, 458030 China; 11 Yangcheng Cancer Hospital, Shanxi, 048100 China; 12 The First People’s Hospital of Ningyang County, Shandong, 271400 China; 13 Cixian Institute for Cancer Prevention and Control, Hebei, 056500 China; 14 Gansu Wuwei Cancer Hospital, Gansu, 733000 China; 15 Minqin County People’s Hospital, Gansu, 733000 China; 16 Linze County People’s Hospital, Gansu, 734200 China; 17 Shandan County People’s Hospital, Gansu, 734000 China; 18 Gaotai County People’s Hospital, Gansu, 734300 China; 19 Department of Gastroenterology, Peking Union Medical College Hospital, Chinese Academy of Medical Sciences, Beijing, 100730 China

**Keywords:** Esophageal squamous cell carcinoma, Precancerous lesions, Latent class analysis, Dietary patterns, Symptom

## Abstract

**Background:**

Dietary patterns and symptoms research among Chinese with esophageal squamous cell carcinoma (ESCC) and its precursor lesions is limited, especially as it relates to multiple food consumption and multiple co-occurring symptoms. The aim of our study was to identify the dietary patterns and severity of symptom classes with the risk of esophageal squamous cell carcinoma and its histological precursor lesions, and develop a risk prediction model for different stages of esophageal disease.

**Methods:**

We analyzed data from a multicenter cross-sectional study carried out in ESCC high incidence areas between 2017 and 2018, which included 34,707 individuals aged 40–69 years. Dietary patterns and severity of symptom classes were derived by applying a latent class analysis (LCA). A multiple logistic regression model was used to derive the odds ratio (ORs) and corresponding 95% confidence intervals (CIs) for ESCC and the different stages of esophageal disease according to the dietary patterns and severity of symptom classes identified. We built the risk prediction model by using a nomogram.

**Results:**

We identified five dietary patterns and three severity of symptom classes. The dietary patterns were classified as follows: “Healthy”, “Western”, “Lower consumers-combination”, “Medium consumers-combination” and “Higher consumers-combination” patterns based on the intake of foods such as red meat, vegetables and fruits. The severity of symptoms was categorized into “Asymptomatic”, “Mild symptoms” and “Overt symptoms” classes based on health-related symptoms reported by the participants. Compared to the “Healthy” pattern, the other four patterns were all associated with an increased risk of esophageal disease. Similarly, the other two symptom classes present different degrees of increased risk of esophageal disease compared to the “Asymptomatic”. The nomograms reflect the good predictive ability of the model.

**Conclusion:**

Among individuals aged 40–69 years in high incidence regions of upper gastrointestinal cancer, the results supplied that subjects with diets rich in livestock and poultry meat and low in fruits and vegetables and subjects with typical symptoms were at increased ESCC risk. The findings highlight the importance of considering food and symptom combinations in cancer risk evaluation.

**Supplementary Information:**

The online version contains supplementary material available at 10.1186/s12885-022-09206-y.

## Background

Esophageal cancer ranks seventh in terms of incidence and sixth in mortality overall in the world according to the report of the Global Cancer Statistics 2020. Eastern Asia shows the highest regional incidence rates for both men and women, partly because of the large burden in China [1]. In China, esophageal cancer is the sixth most common malignancy and the fourth leading cause of cancer-related deaths [2]. Esophageal adenocarcinoma (EADC) and esophageal squamous cell carcinoma (ESCC) are the two most common histologic subtypes of esophageal cancer. In China, more than 90% of esophageal cancer cases are ESCCs, whose number accounts for about half of all the ESCC cases on earth [3]. The most surveyed region of China is the North Central Taihung Mountain range. In small areas of this region, ESCC may be at or nearly the leading cause of death, with incidence rates exceeding 125/100,000 per year [4]. Therefore, identification of risk factors in the early stages of the disease appears to be essential in order to decrease ESCC incidence and mortality.

Esophagitis is a precancerous disease of esophageal cancer, and it is a benign disease with a certain canceration rate. Based on WHO tumor histological classification, the esophageal precancerous lesions (EPL) can be defined as low-grade intraepithelial neoplasia (LGIN) and high-grade intraepithelial neoplasia (HGIN) [5]. With the aggravation of precancerous lesions, the rate of developing esophageal cancer increased from 24 to 74% [6]. Therefore, intervention at an early stage of the disease results in a significant decrease in ESCC incidence and mortality. The etiology of ESCC is multi-factorial and strongly population dependent. A study estimated a population-attributable risk of 89% using only cigarette smoking, alcoholic beverage consumption, and low consumption of fruits and vegetables [7]. There is also some evidence on the protective effect of fruit and vegetable and the potential harmful effect of processed/red meat consumption. The evidence on the effect of diet on ESCC risk is however still suggestive or limited [8]. Before a diagnosis of esophageal cancer is established, majority of the patients have experienced pain, dysphagia, eating difficulties, appetite loss, bloating and nausea resulting in patients’ daily living and quality of life [9]. Therefore, it is more meaningful for the prevention of esophageal cancer that identifying the high risk dietary patterns and some classical symptoms in the early stage of esophageal cancer.

The analysis of dietary patterns aims to fully explore the complexity of the diet, as an alternative to the study of isolated components. These techniques depend upon the concept that food consumption can be effectively presented by reproducible patterns, in spite of individual variations, and that food eaten together may have interactive effects on the risk of cancer. Similarly, the symptoms are the same. Identifying symptom clusters and their relationship to patient characteristics may lead to a better interpretation for identifying patients with early lesions and provide greater insight into the planning of future interventions.

Latent class analysis (LCA) is a model-based cluster analysis technique that allows for identifying prevalent, mutually exclusive, dietary patterns and symptoms with additional advantages with respect to the classical approaches [10]. Unlike principal component analysis (PCA) and factor analysis (FA), it can be used to categorize individuals into mutually exclusive groups, dietary patterns or severity of symptoms and differently from cluster analysis, it grants quantification of the uncertainty of class membership, and assessment of goodness of fit [11].

Our study aims to identify dietary patterns and severity of symptom through LCA, and thus to model screening for different stages of the disease. Therefore, adding a new perspective on the association between dietary habits and symptoms and ESCC in China.

## Methods

### Study population

This was a multicenter cross-sectional study, depending on high incidence regions of esophageal and gastric cancer established by the cancer early diagnosis and early treatment project in some high risk areas in China since 2000 [12]. In 2017, a new screening study of upper gastrointestinal cancer in five high-risk rural areas in China, including Hebei, Henan, Shandong, Shanxi, and Gansu Provinces was released. Men and women age 40 to 69 years were all selected as the target population. The main purpose of this project was to confirm the high-risk population of malignant upper gastrointestinal cancer and to establish a cancer risk prediction model to provide support for the prevention of upper gastrointestinal cancer.

**Fig. 1:**
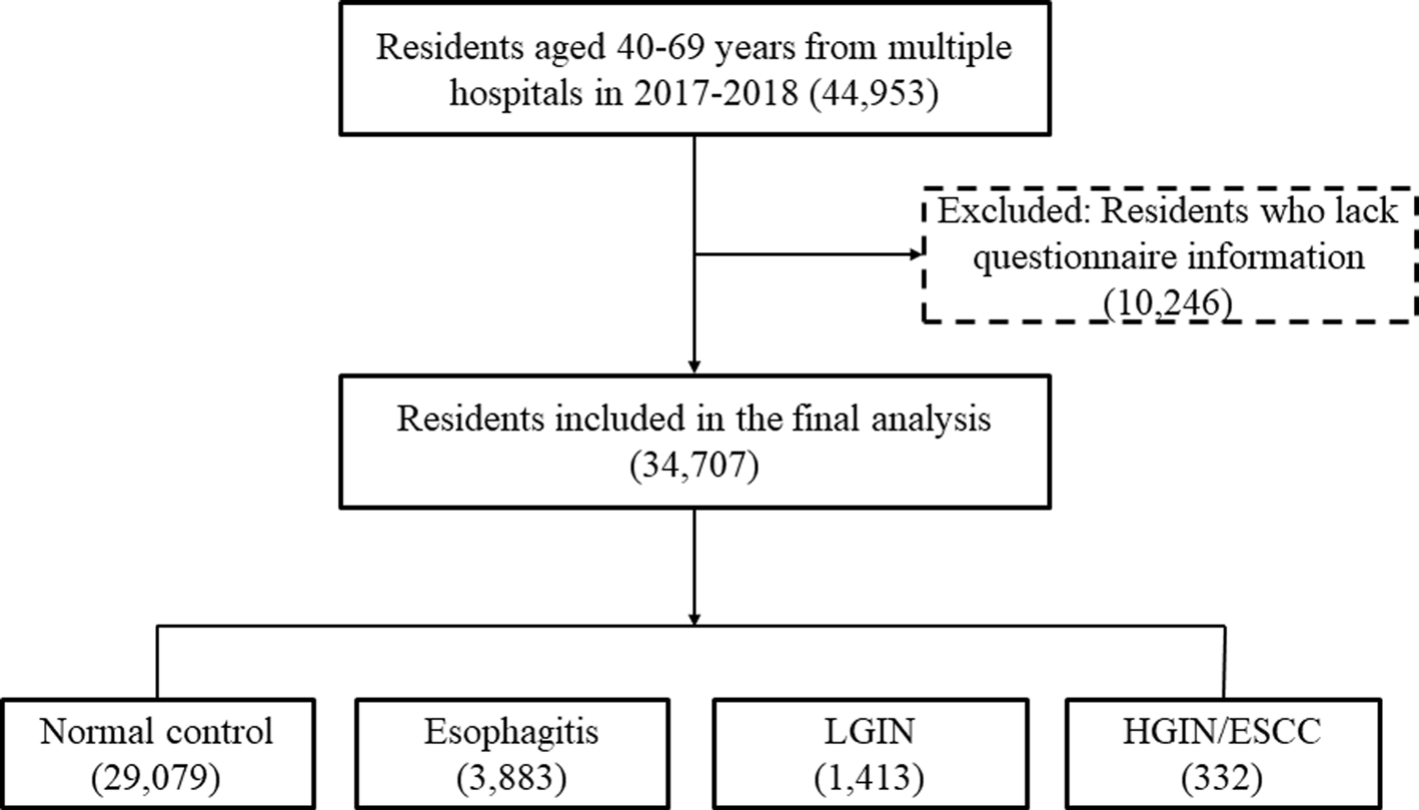
Flow chart of participant selection. Note: LGIN = low-grade intraepithelial neoplasia; HGIN = high-grade intraepithelial neoplasia; ESCC = esophageal squamous cell carcinoma

The inclusion criteria were as follows: (1) local permanent residents in selected regions, (2) no history of endoscopic examination during the last 3 years, (3) no history of cancer, mental disorder, or any contraindication for endoscopy, (4) signed informed consent and (5) agreement to complete the entire survey and examination, including endoscopy. The participant selection process is shown in Fig. 1. We recruited participants from April 2017 to December 2018. The final analysis included 34,707 residents aged 40–69 years. Among these participants, there were 81 persons with ESCC, 251 persons with HGIN, 1413 persons with LGIN, 3883 persons with esophagitis and 29,079 persons serving as normal esophagus controls.

The study was approved by the Capital Medical University, Chinese Academy of Medical Sciences and Peking Union Medical College. The experimental protocol involving humans was in accordance to the guidelines of the Declaration of Helsinki.

### Diet and symptoms assessment

Comprehensive questionnaire information was collected by face-to-face interviews and entered directly into a laptop based data entry system by trained investigators. The data entry process was conducted with software designed to decrease missing items and reduce logic inaccuracy. A questionnaire typically took 35–45 min to complete. Items of dietary intake were selected through the above questionnaire, including livestock meat and its products (cured, processed and salted meats), poultry meat, seafood, eggs and their products, vegetables, fruits, bean products, scallions, ginger and garlic, pickles and nuts. All the variables were categorical. Foods that are consumed more frequently, were divided into three categories namely: every day, 1–6 days per week, and less than 1 day per week. Foods that are consumed infrequently, were divided into two categories namely: at least one day per week and less than one day per week. Items of typical patient-reported symptoms were also selected through the above questionnaire, including number of lost teeth, frequent bleeding of gums, dysphagia, bloating, heartburn, acid reflux, nausea, vomiting, belching and epigastric pain. The number of lost teeth were categorized into three groups namely: never dropped, 1–3 teeth and more than 4 teeth. Other variables are categorized as yes or no.

### Outcome assessment

The endoscopic examinations were carried out by physicians at local hospitals. Procedures were based on clinical guidelines for cancer screening and early diagnosis and treatment in China. Lugol’s iodine staining method was used to identify suspicious tissues, which were then biopsied. To confirm severity, the esophageal mucosa was ranked into 5 categories: normal esophageal mucosa, minor mucosa changes, esophagitis, esophageal squamous simple hyperplasia (ESSH) or esophageal squamous dysplasia (ESD) [13]. ESD was further classified into 3 levels including slight, moderate, and severe. According to WHO tumor histological classification, mild and moderate ESD combined fall under LGIN. Severe ESD and squamous cell carcinoma in situ are considered as HGIN. If there were any inconsistencies, a third pathologist would give advice through discussion. Doctors reported the worst biopsy diagnosis to be from participants with multiple lesions. In this study, we divided the participants into 4 groups: normal control, esophagitis, LGIN and HGIN/ESCC.

### Statistical methods

We characterized the dietary patterns and symptom patterns, assumed as unobserved mutually exclusive, with different variables probability distributions, by performing LCA on the observed responses on the different items.

LCA identified latent classes of participants based on the ten dietary variables and six symptom variables. Estimation was conducted with the robust maximum-likelihood and expectation-maximization algorithms [14]. Statistical fit indexes were used to assess model fit and to decide the final number of latent classes. The model that fits the data best was selected by a combination of the following criteria: (a) the lowest Akaike information criterion (AIC), (b) the lowest Bayesian information criterion (BIC), (c) the lowest Lo–Mendell–Rubin likelihood ratio test (LMR), (d) the lowest Lo–Mendell–Rubin Adjusted LMR test (ALMR), and (e) entropy to be 0.6 or greater [15]. Next, we executed an unconditional multivariable logistic regression to identify sociodemographic and risk factors that predicted class membership. Before conducting the analysis, we performed a covariance diagnosis between the independent variables. We considered models to calculate the adjusted odds ratios (ORs) and 95% confidence intervals (CIs), including age, gender, education, body mass index (BMI), smoking and drinking at the same time. We also used a nomogram to model normal controls separately from the different stages of the disease. Evaluation of the model was performed using calibration curves and decision curves. In addition, basic, descriptive statistics show categorical variables as percentages, while continuous variables are shown as mean and standard deviations.

LCA was conducted in both cases and normal controls. Analysis of only the normal control was performed to check the robustness of the previous solution. As dietary patterns and symptom severity classes identified on controls were consistent (number and characteristics of the patterns) with the ones obtained on the overall dataset, we based all our analysis on the overall dataset. To guarantee the internal reproducibility of the chosen solution the analysis was conducted separately in two randomly selected subsets of the original data several times.

Statistical analyses were performed using Mplus (version 8.1) and R (version 3.6.3) software. All tests were two-sided and had a significance level of 0.05.

## Results

When fitting the LCA model, we selected the result with 5 classes for dietary patterns and 3 classes for the severity of symptom in every group according to the criteria in methods (Tables 1 and 2).

**Table 1:** Model fit information for latent class analysis of dietary patterns

Subtypes	Model	AIC	BIC	Entropy	LMR *P*-value	ALMR *P*-value
Overall	1-Class	403,812.534	403,930.899			
	2-Class	386,659.699	386,904.886	0.597	0.0000	0.0000
	3-Class	380,960.196	381,332.203	0.628	0.0000	0.0000
	4-Class	377,988.054	378,486.881	0.672	0.0000	0.0000
	5-Class	376,775.273	377,400.920	0.648	0.0000	0.0000
	6-Class	376,013.990	376,766.458	0.630	0.0499	0.0506
	7-Class	375,382.845	376,262.134	0.616	1.0000	1.0000
Esophagitis	1-Class	383,546.414	383,664.057			
	2-Class	367,382.696	367,626.386	0.596	0.0000	0.0000
	3-Class	361,925.857	362,295.594	0.629	0.0000	0.0000
	4-Class	359,187.987	359,683.771	0.671	0.0000	0.0000
	5-Class	358,056.094	358,677.925	0.648	0.0000	0.0000
	6-Class	357,318.205	358,066.082	0.632	1.0000	1.0000
LGIN	1-Class	354,511.583	354,628.136			
	2-Class	339,894.092	340,135.524	0.590	0.0000	0.0000
	3-Class	335,082.275	335,448.584	0.619	0.0000	0.0000
	4-Class	332,591.286	333,082.474	0.662	0.0000	0.0000
	5-Class	331,522.734	332,138.800	0.638	0.0000	0.0000
	6-Class	330,813.727	331,554.671	0.623	0.0666	0.0674
	7-Class	330,280.873	331,146.696	0.605	0.7604	0.7604
HGIN and ESCC	1-Class	341,936.596	342,052.644			
	2-Class	327,933.965	328,174.349	0.588	0.0000	0.0000
	3-Class	323,291.949	323,656.671	0.620	0.0000	0.0000
	4-Class	321,000.163	321,489.221	0.659	0.0000	0.0000
	5-Class	319,970.157	320,583.552	0.638	0.0000	0.0000
	6-Class	319,270.147	320,007.879	0.626	0.0687	0.0696
	7-Class	318,754.107	319,616.176	0.605	0.5446	0.5464

Note: *LGIN* low-grade intraepithelial neoplasia, *HGIN* high-grade intraepithelial neoplasia, *ESCC* esophageal squamous cell carcinoma, *AIC* akaike information criterion, *BIC* bayesian information criterion, *LMR* Lo–Mendell–Rubin likelihood ratio test, *ALMR* Lo–Mendell–Rubin Adjusted LMR test

**Table 2:** Model fit information for latent class analysis of symptom classes

Subtypes	Model	AIC	BIC	Entropy	LMR *P*-value	ALMR *P*-value
Overall	1-Class	136,196.132	136,255.315			
	2-Class	134,911.554	135,038.375	0.898	0.0000	0.0000
	3-Class	134,613.394	134,807.852	0.935	0.0000	0.0000
	4-Class	134,595.780	134,857.876	0.623	0.1470	0.1508
Esophagitis	1-Class	129,391.888	129,450.710			
	2-Class	128,168.972	128,295.018	0.896	0.0000	0.0000
	3-Class	127,893.905	128,087.176	0.821	0.0000	0.0000
	4-Class	127,881.486	128,141.983	0.620	0.5484	0.5522
LGIN	1-Class	120,040.246	120,098.523			
	2-Class	118,822.702	118,947.580	0.890	0.0000	0.0000
	3-Class	118,587.299	118,778.779	0.932	0.0000	0.0000
	4-Class	118,569.312	118,827.393	0.648	0.0750	0.0778
HGIN and ESCC	1-Class	115,838.134	115,896.158			
	2-Class	114,651.925	114,776.262	0.889	0.0000	0.0000
	3-Class	114,426.629	114,617.279	0.850	0.0000	0.0000
	4-Class	114,414.785	114,671.748	0.688	0.1674	0.1721

Note: *LGIN* low-grade intraepithelial neoplasia, *HGIN* high-grade intraepithelial neoplasia, *ESCC* esophageal squamous cell carcinoma, *AIC* akaike information criterion, *BIC* bayesian information criterion, *LMR* Lo–Mendell–Rubin likelihood ratio test, *ALMR* Lo–Mendell–Rubin Adjusted LMR test

### Latent classes of dietary patterns

Table 3 shows the conditional distribution of food group intake for all participants, giving the latent classes for the food groups that were more relevant in discriminating and labeling the clusters. The table of the normal control and the other three groups of different stages of disease are given in the Supporting Information Table S1-S3. We identified five classes with similar probability distributions according to a previous study [10], both for all participants and for different stages of the disease. Cluster 1 labeled “Healthy pattern”, showed higher probability to consume more fruiting vegetables and all other kinds of fruits, high-quality protein and lower probability to consume red meat. Subjects in Cluster 2, “Western pattern,” reported higher consumption of red meat and lower consumption of vegetables and fruits. Clusters 3 to 5 were related to previous food groups, but with a difference in the amount of intake. We termed Cluster 3 “Lower consumers-combination pattern” as people in it were less likely to eat all kinds of food. Cluster 4 had slightly higher probability than cluster 3 to eat all kinds of food. We called this cluster “Medium consumers-combination pattern.” Cluster 5 had the highest probability as compared to cluster 3 and 4 to eat all kinds of food, so we name it as “Higher consumers-combination pattern.” The estimated cluster sizes were 28.8% (*n* = 9990) for the “Healthy pattern,” 9.3% (*n* = 3216) for the “Western pattern,” 29.1% (*n* = 10,100) for the “Lower consumers-combination pattern,” 28.7% (*n* = 9971) for the “Medium consumers-combination pattern” and 4.1% (*n* = 1430) for the “Higher consumers-combination pattern.”

**Table 3:** Probabilities of consumption for selected food items by dietary patterns derived from LCA for all participants

Food items		Healthy	Western	Lower consumers-combination	Medium consumers-combination	Higher consumers-combination
Livestock meat and its products	everyday	77.1%	84.7%	14.8%	9.8%	54.0%
	1-6 days/week	12.5%	12.6%	22.3%	72.2%	43.3%
	< 1 day/week	10.4%	2.7%	62.9%	18.0%	2.7%
Poultry meat	≥1 day/week	7.0%	7.4%	1.5%	3.3%	89.1%
	< 1 day/week	93.0%	92.6%	98.5%	96.7%	10.9%
Seafood	≥1 day/week	1.5%	9.1%	1.5%	0.3%	64.8%
	< 1 day/week	98.5%	90.9%	98.5%	99.7%	35.2%
Eggs and its products	everyday	61.3%	73.1%	22.0%	31.9%	50.1%
	1-6 days/week	15.2%	16.4%	24.1%	64.5%	38.4%
	< 1 day/week	23.5%	10.5%	53.9%	3.6%	11.5%
Vegetables	everyday	99.8%	0.0%	2.5%	2.7%	97.2%
	1-6 days/week	0.1%	0.6%	2.6%	97.3%	2.3%
	< 1 day/week	0.1%	99.4%%	94.9%	0.0%	0.5%
Fruits	everyday	41.4%	18.6%	3.0%	2.8%	31.8%
	1-6 days/week	21.0%	41.4%	11.9%	59.5%	54.8%
	< 1 day/week	37.6%	40.0%	85.1%	37.7%	13.4%
Bean products	≥1 day/week	38.0%	46.3%	8.6%	45.3%	65.7%
	< 1 day/week	62.0%	53.7%	91.4%	54.7%	34.3%
Scallion, ginger and garlic	≥1 day/week	93.7%	72.7%	36.6%	59.9%	84.7%
	< 1 day/week	6.3%	27.3%	63.4%	40.1%	15.3%
Pickles	≥1 day/week	4.8%	68.8%	3.1%	11.7%	17.9%
	< 1 day/week	95.2%	31.2%	96.9%	88.3%	82.1%
Nut fruits	≥1 day/week	16.9%	33.8%	2.1%	5.0%	24.2%
	< 1 day/week	83.1%	66.2%	97.9%	95.0%	75.8%
Cluster’s size		28.8%	9.3%	29.1%	28.7%	4.1%

Descriptions of the clusters for selected variables are given in Table 4. The proportion of smokers and drinkers was higher in the Western pattern compared to the Healthy pattern. For the other three patterns, the proportion of smokers to drinkers gradually increased with increasing food consumption. A similar trend was shown in the other three subgroups (data not shown).

**Table 4:** Dietary patterns’ characteristics according to selected sociodemographic variables for all participants

Characteristics		Healthy	Western	Lower consumers-combination	Medium consumers-combination	Higher consumers-combination
Population groups (male:%)	Normal control (39.8%)	89.1%	84.0%	79.7%	82.4%	84.1%
	Esophagitis (45.8%)	6.9%	9.7%	14.7%	12.6%	10.4%
	LGIN (47.0%)	3.3%	5.1%	4.5%	4.0%	4.5%
	HGIN and ESCC (52.7%)	0.7%	1.2%	1.1%	1.0%	1.0%
Age	40–50	33.4%	28.0%	27.4%	33.1%	33.8%
	51–60	44.3%	45.3%	42.6%	41.6%	41.3%
	61–69	22.3%	26.7%	30.0%	25.3%	24.9%
Gender	Male	41.3%	46.4%	40.0%	38.9%	46.0%
	Female	58.7%	53.6%	60.0%	61.1%	54.0%
Education	Never been to school	14.9%	13.3%	16.1%	14.5%	12.0%
	Primary school	34.3%	33.0%	39.6%	35.4%	30.8%
	Middle school	39.8%	43.9%	35.6%	38.7%	42.2%
	High school and above	11.0%	9.8%	8.7%	11.4%	15.0%
BMI	< 18.5	1.8%	2.0%	1.8%	1.9%	2.0%
	18.5–23.9	46.6%	44.1%	48.9%	44.7%	48.3%
	24.0–27.0	39.7%	40.1%	39.4%	40.9%	39.7%
	> 27.0	11.9%	13.8%	9.9%	12.5%	10.1%
Smoking status	Never smoked	74.9%	64.5%	77.5%	77.5%	73.2%
	Current smoking	20.6%	27.1%	19.9%	18.4%	22.0%
	Previous smoking	4.5%	8.4%	2.7%	4.1%	4.8%
Drinking status	No alcohol consumption	62.0%	57.6%	75.6%	63.9%	64.3%
	< 1 time/week	28.8%	20.1%	19.5%	29.1%	20.0%
	≥1 time/week	9.2%	22.3%	4.95%	7.0%	15.7%

Note: *LGIN* low-grade intraepithelial neoplasia, *HGIN* high-grade intraepithelial neoplasia, *ESCC* esophageal squamous cell carcinoma, *BMI* body mass index

### Latent classes of severity of symptom

Table 5 shows the conditional distribution of symptom group for all participants, giving the latent classes for the symptom groups more relevant in discriminating and labeling the clusters. The table of the normal control and the other three groups of the different stages of disease are given in the Supporting Information Table S4-S6. For all participants and for different stages of the disease, there were three classes with similar probability distributions. We named the first class “Asymptomatic” as subjects were reported to be relatively healthy and not showing any symptoms. Class 2 was named “Mild symptoms” as subjects in this cluster reported significant symptoms in terms of gingival bleeding. Subjects in the last class had a high percentage of symptoms reported in all areas. We named this class “Overt symptoms.” Sizes of the severity of symptom classes were 71.9% for the “Asymptomatic”, 26.7% for the “Mild symptoms” and 1.5% for the “Overt symptoms.”

**Table 5:** Probabilities for selected symptom items by severity of symptom derived from LCA for all participants

Symptom items		Asymptomatic	Mild symptoms	Overt symptoms
Number of lost teeth	0 teeth	48.4%	39.5%	32.8%
	1–3 teeth	29.5%	39.3%	31.6%
	more than 4 teeth	22.1%	21.1%	35.6%
Whether gingival bleeding	Yes	0.0%	100.0%	35.1%
	No	100.0%	0.0%	64.9%
Whether dysphagia	Yes	0.2%	0.1%	7.0%
	No	99.8%	99.9%	93.0%
Whether bloating, heartburn, acid reflux	Yes	1.6%	1.4%	47.6%
	No	98.4%	98.6%	52.4%
Whether nausea, vomiting and belching	Yes	0.6%	1.0%	36.6%
	No	99.4%	99.0%	63.4%
Whether epigastric pain	Yes	0.8%	0.8%	20.1%
	No	99.2%	99.2%	79.9%
Cluster’s size		71.9%	26.7%	1.5%

Further description of the severity of symptom classes for a selected set of variables are shown in Table 6. Differences between the clusters in demographics were not particularly significant. A similar trend was shown in the other three subgroups (data not shown).

**Table 6:** Severity of symptoms’ characteristics according to selected sociodemographic variables for all participants

Characteristics		Asymptomatic	Mild symptoms	Overt symptoms
Population groups (male:%)	Normal control (39.8%)	84.0%	82.8%	89.1%
	Esophagitis (45.8%)	11.0%	11.9%	5.9%
	LGIN (47.0%)	4.0%	4.5%	3.8%
	HGIN and ESCC (52.7%)	1.0%	0.8%	1.2%
Age	40–50	30.3%	33.5%	25.9%
	51–60	43.1%	43.0%	40.5%
	61–69	26.6%	23.5%	33.6%
Gender	Male	41.9%	38.5%	36.6%
	Female	58.1%	61.5%	63.4%
Education	Never been to school	15.0%	13.8%	25.5%
	Primary school	35.0%	38.4%	33.6%
	Middle school	39.0%	38.4%	29.4%
	High school and above	11.0%	9.4%	11.5%
BMI	< 18.5	1.8%	1.8%	4.0%
	18.5–23.9	46.0%	47.8%	51.0%
	24.0–27.0	40.1%	39.9%	36.4%
	> 27.0	12.1%	10.5%	8.7%
Smoking status	Never smoked	74.2%	78.5%	74.9%
	Current smoking	21.5%	17.6%	19.0%
	Previous smoking	4.3%	3.9%	6.1%
Drinking status	No alcohol consumption	65.5%	67.9%	70.6%
	< 1 time/week	24.6%	26.0%	25.7%
	≥1 time/week	9.9%	6.1%	3.8%

Note: *LGIN* low-grade intraepithelial neoplasia, *HGIN* high-grade intraepithelial neoplasia, *ESCC* esophageal squamous cell carcinoma, *BMI* body mass index

### Logistic regression analysis and nomogram

We applied an unconditional multivariable logistic regression to analyze dietary patterns as well as symptom severity for normal controls vs different stages of esophageal diseases. The results of the covariance diagnosis showed no significant covariance between the independent variables (data not shown). Tables 7 and 8 shows the ORs and 95%CI for all stages of disease, by the classification in the five dietary patterns and three symptom severity classes from the composite model including the relevant confounding and risk variables. In the dietary patterns, compared to the “Healthy” pattern, the “Western”, the “Lower consumers-combination”, the “Medium consumers-combination” and the “Higher consumers-combination” were positively related to the risk of the progression of the disease stage for esophagitis (OR = 1.42, 95%CI: 1.23–1.53; OR = 2.33, 95%CI: 2.11–2.57; OR = 1.99, 95%CI: 1.80–2.19 and OR = 1.59, 95%CI: 1.32–1.92, respectively). Consistent results were also observed between the normal control, LGIN/HGIN and ESCC three groups. The “Western”, the “Lower consumers-combination”, the “Medium consumers-combination” and the “Higher consumers-combination” patterns showed a positive association with the risk of LGIN and HGIN and ESCC compared to the “Healthy” pattern. In the symptom severity classes, compared to the “Asymptomatic” class, the “Mild symptoms” class was positively related to the risk of the progression of the disease stage for the esophagitis and LGIN groups, OR = 1.87, 95%CI: 1.66–2.10, OR = 1.25, 95%CI: 1.11–1.41 for the “Mild symptoms” class and OR = 1.04, 95%CI: 0.86–1.25 and OR = 0.82, 95%CI: 0.51–1.31 for the “Overt symptoms” class. However, in the HGIN&ESCC group, the “Overt symptoms” class was positively related to the risk of HGIN&ESCC compared to the “Asymptomatic” class (OR = 1.58, 95%CI: 1.00–2.50), The “Mild symptoms” class did not differ significantly from the “Asymptomatic” class (OR = 0.98, 95%CI: 0.58–1.66).

**Table 7:** Logistic regression analysis of associations between dietary patterns with esophageal cancer and precancerous lesions

Dietary Patterns	Esophagitis		LGIN		HGIN and ESCC	
	N (%)	OR(95%CI)	N (%)	OR(95%CI)	N (%)	OR(95%CI)
Healthy	684(17.6)	1.0	320(22.6)	1.0	64(19.3)	1.0
Western	314(8.1)	1.42(1.23–1.53)	173(12.2)	1.54(1.27–1.87)	42(12.7)	1.69(1.14–2.52)
Lower consumers-combination	1483(38.2)	2.33(2.11–2.57)	444(31.4)	1.39(1.20–1.61)	109(32.8)	1.72(1.26–2.36)
Medium consumers-combination	1252(32.3)	1.99(1.80–2.19)	411(29.1)	1.30(1.12–1.51)	102(30.7)	1.64(1.19–2.24)
Higher consumers-combination	149(3.8)	1.59(1.32–1.92)	65(4.6)	1.44(1.09–1.90)	15(4.5)	1.62(0.92–2.86)

Note: *LGIN* low-grade intraepithelial neoplasia, *HGIN* high-grade intraepithelial neoplasia, *ESCC* esophageal squamous cell carcinoma, *OR* odds ratio, *CI* confidence interval

**Table 8:** Logistic regression analysis of associations between symptom clusters with esophageal cancer and precancerous lesions

Severity of symptom	Esophagitis		LGIN		HGIN and ESCC	
	N (%)	OR(95%CI)	N (%)	OR(95%CI)	N (%)	OR(95%CI)
Asymptomatic	3367(86.7)	1.0	979(69.3)	1.0	297(89.5)	1.0
Mild symptoms	378(9.7)	1.87(1.66–2.10)	415(29.4)	1.25(1.11–1.41)	15(4.5)	0.98(0.58–1.66)
Overt symptoms	138(3.6)	1.04(0.86–1.25)	19(1.3)	0.82(0.51–1.31)	20(6.0)	1.58(1.00–2.50)

Note: *LGIN* low-grade intraepithelial neoplasia, *HGIN* high-grade intraepithelial neoplasia, *ESCC* esophageal squamous cell carcinoma, *OR* odds ratio, *CI* confidence interval.

**Fig. 2:**
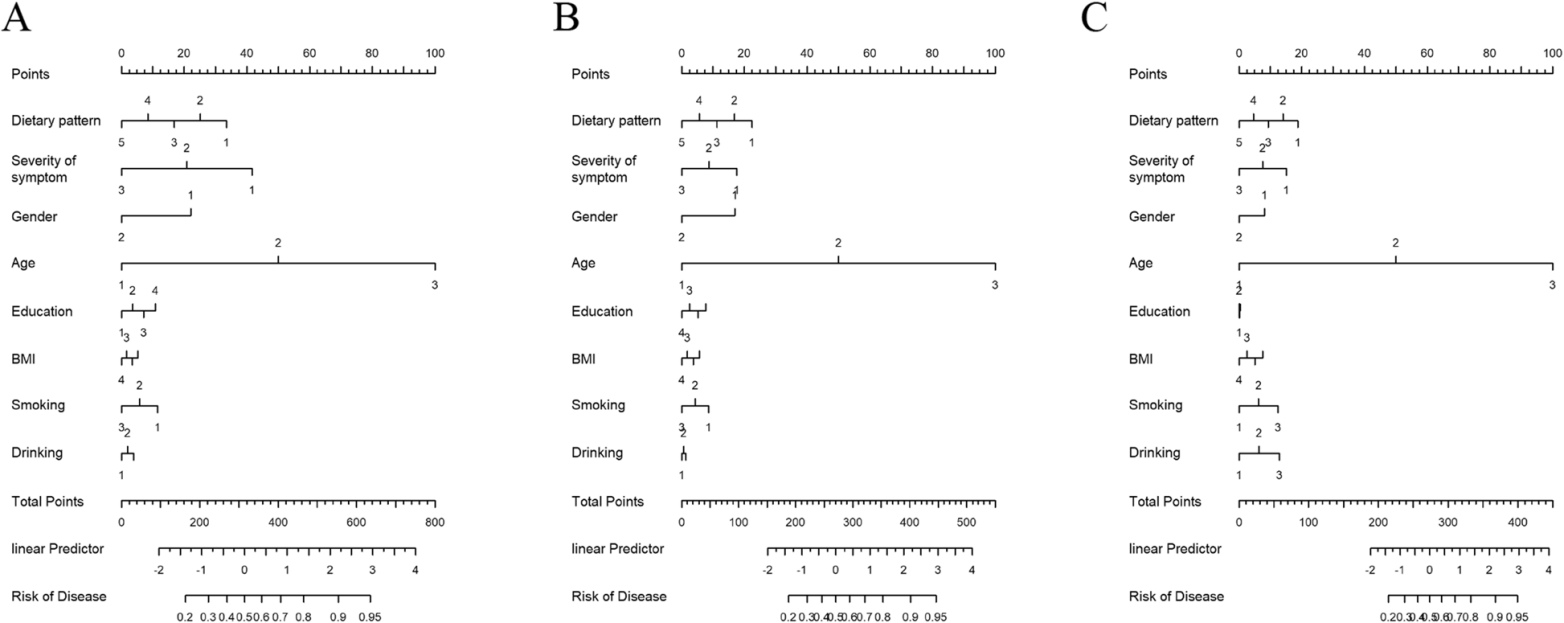
**A**: Nomogram predicting the risk for the normal control group vs esophagitis; **B**: Nomogram predicting the risk for the normal control group vs LGIN; **C**: Nomogram predicting the risk for the normal control group vs HGIN and ESC. Note: LGIN: low-grade intraepithelial neoplasia; HGIN: high-grade intraepithelial neoplasia; ESCC: esophageal squamous cell carcinoma

**Fig. 3:**
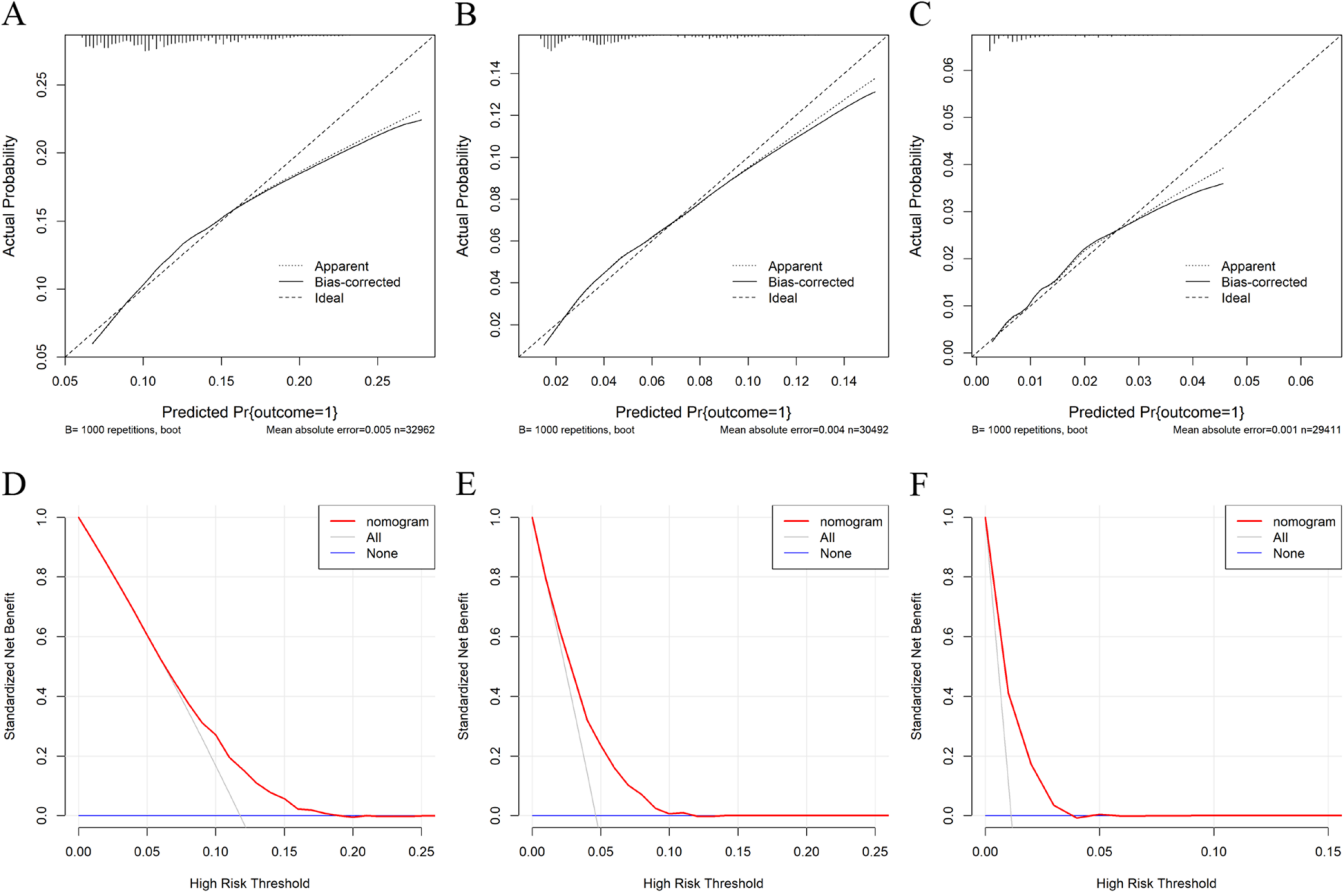
**A**-**C**: Calibration curves showing the probability of the normal control vs esophagitis/LGIN/HGIN and ESCC three groups between the nomogram prediction and the actual observation. Perfect prediction would correspond to a slope of 1 (diagonal 45-degree gray line). **D**-**F**: Decision curves of the nomogram predicting the risk. The x-axis represents the threshold probabilities, and the y-axis measures the net benefit calculated by adding the true positives and subtracting the false positives. Note: LGIN: low-grade intraepithelial neoplasia; HGIN: high-grade intraepithelial neoplasia; ESCC: esophageal squamous cell carcinoma

After logistic regression, we performed nomogram building and internal validation for each of the three subgroups. The model was virtually presented in the form of a nomogram (Fig. 2), the C-index of the novel nomogram was 0.612, 0.684 and 0.746, respectively for esophagitis, LGIN and HGIN&ESCC groups, embodying the good predictive ability of the model. The calibration curves also showed good consistency in the probability between the actual observation and the nomogram prediction (Fig. 3 a-c). In addition, decision curve analyses (DCA) exhibited great positive net benefits in the predictive model among almost all of the threshold probabilities at different groups, indicating the favorable potential clinical effect of the predictive model (Fig. 3 d-f).

## Discussion

This study is the first latent class analysis based on a natural population in a high incidence area of esophageal cancer in China. Additionally, this is the largest study to use latent class analysis to describe multiple dietary patterns and symptoms experienced by high incidence areas of esophageal cancer in China. In our study, we identify dietary patterns and severity of symptoms conceived as mutually exclusive groups of people characterized by similar food intake and symptom clusters and to compare the resulting classes in terms of different stages of disease risk.

Dietary factors as well as early symptoms are considered of significantly influencing ESCC risk. Recent evidence came from studies focusing on single foods [16, 17] or single symptom [18–23], rather than dietary patterns and symptom clusters. Here, we used latent class analysis to explore relationships between ESCC risk and dietary patterns characterized by varying levels of fruit, vegetable, and red meat intake in multicenter. Meanwhile, we also investigate the relationships between symptom cluster and risk of ESCC characterized by different levels of lost teeth, gingival bleeding, and some specific symptoms Most of the previous studies on the dietary pattern of esophageal cancer have been conducted only for esophageal cancer and not for precancerous lesions [24]. Our study focused not only on ESCC cases, but also on the precancerous lesions with different dietary patterns, which is more likely to provide individualized dietary recommendations for the early prevention of ESCC.

We classified five dietary patterns. We discovered a protective effect on ESCC risk for a diet rich in vegetables and fruit consumption and associated with a low intake of red meat and pickles (Healthy pattern). Compared to this group, the “Western” pattern, distinguished by a low consumption of fruit and vegetables and high intake of red meat and pickles, was associated with an increased risk of esophagitis, LGIN, HGIN and ESCC. The“Lower consumers-combination” pattern, which showed a diet deficient in most of the foods considered, was positively related to the different stages of disease risk. The higher caloric intake of the “Higher consumers-combination” pattern, leads to an increased risk of the different stages of disease. And the “Medium consumers-combination” pattern is somewhere in between.

Our results are consistent with most of the studies evaluating the influence of diet on ESCC risk [24]. The pattern with a high intake of fruit and vegetables, named “Healthy” or “Vegetable and Fruit” was found and associated with a decrease of ESCC risk in most of these studies [25–28]. High dietary fiber intake has also been found to significantly reduce the risk for Barrett’s esophagus and esophageal cancer [29]. As previous study suggested, inositol hexaphosphate, one component in food sources high in dietary fiber, has been proven in vitro experiment to inhibit the growth rate of esophageal cancer cells by decreasing cellular proliferation and stimulating apoptosis [30]. Dietary fiber phenolic compounds ferulic acid and pcoumaric acid may have an anti-proliferative effect on cell cycle [31]. In addition, a high dietary fiber diet is associated with lower plasma levels of systematic inflammation biomarkers such as tumor necrosis factor-α receptor-2 (TNF-α-R2) and interleukin-6 (IL-6), which may influence the process of carcinogenesis [32].

A potential adverse effect of a diet mainly based on red meat, processed food in general was also found [33, 34]. In a previous study, despite different labelling, number of components and weighting, the most commonly classified patterns were an ‘unhealthy’ one (often named ‘Western’) and a ‘healthy’ one in different country [35]. The other three patterns were not common in previous studies. The“Lower consumers-combination” pattern, is usually composed of people whose dietary intake is insufficient. Previous studies have reported a significantly increased risk of esophageal cancer in malnourished populations [36]. For the “Higher consumers-combination” pattern, the increased risk of esophageal cancer may be due to the high caloric intake of food. And a case-control study conducted in an Iranian population showed that higher intake of calories and total fat significantly increased the risk of esophageal cancer [37]. The increased risk of esophageal cancer in the “Medium consumers-combination” pattern may be due to the high intake of red meat and processed foods and relatively low intake of fruits and vegetables. The reasons for similar results in the subgroup analysis can be explained in the same way.

We classified three symptom severity classes defined as mutually exclusive groups of subjects characterized by different symptoms. We found that at the stage of esophagitis and LGIN, the patient has only mild symptoms, while at the stage of HGIN and ESCC, the patient has relatively severe symptoms. If symptoms can be identified early in the disease process, the development of the disease can be reduced. In esophagitis and in the LGIN stage, compared to the “Asymptomatic” class, the “Mild symptoms” class may experience tooth loss and frequent gingival bleeding. In a study of risk factors for esophageal cancer and its precancerous lesions conducted in Henan, China, a high number of missing teeth was found to be a significant risk factor [38]. In another studies of a Chinese population, tooth loss was found to be a risk factor for esophageal squamous cell cancer [19] as well as gastric cancer [39]. The hypothesis, frequently cited in the ESCC etiology literature, is that incomplete chewing and rapid swallowing of large pieces of food might lead to irritation or damage to the esophageal epithelium and subsequently increase the risk of ESCC [40]. In the current study, the relationship between the number of teeth lost and ESCC risk supports this hypothesis. Another hypothesis is that poor oral hygiene and tooth loss mediate a bacterial load and “overgrowth” of microorganisms on teeth [41], which can transform nitrates into nitrites and then combine with amines to form carcinogenic nitrosamines, some of which may be gastrointestinal organ-specific carcinogens [42, 43]. Therefore, an association between tooth loss and esophageal disease seems plausible. For people with frequent gingival bleeding, the increased risk of esophageal disease may be due to poor oral health as a result of bleeding gums. And many studies have shown that poor oral health is a risk factor for the development of esophageal cancer [44–47]. In the HGIN and ESCC group, compared to the “Asymptomatic” class, the “Overt symptoms” class has an increased risk of HGIN and ESCC. In this class, each symptom has a certain percentage of occurrence, especially upper gastrointestinal symptoms such as dysphagia, nausea, vomiting, etc. This indicates that upper gastrointestinal symptoms are already present when esophageal squamous cell carcinoma occurs or is about to occur. This reinforces the importance of intervention at an early stage of the disease.

LCA can bring interesting insights into dietary patterning and symptom severity classes, allowing to identify prevalent types of eating behavior and severity of symptom in a population and to compare risk for people with different types of diet and symptom severity. Traditional methods such as PCA and FA, which have the disadvantage of not being able to produce mutually exclusive groups. Thus, when the interest is to compare subgroups of subjects, an additional step of cross-classification of the dimensions/combinations is needed. The application of a LCA to this study on different stages of esophageal disease overcomes problems inherent in the traditional methods and gives further advantages in dietary patterning and symptom severity class, such as a probability-based classification under a general parametric approach and pattern prevalence estimation.

A nomogram is a convenient tool to anticipate and quantify the chance of an individual patient progressing to a certain clinical event. Nomograms are helpful in clinical decision making and valuable in risk stratification and individualized treatment [48]. Based on the nomogram, we can calculate the individual risk score and thus estimate the individual risk of disease. The identification of individuals as patients with early-stage disease, coupled with timely interventions, leads to a decrease in the incidence of ESCC. In this study, the nomogram we created had a good predictive power. Using, age, gender, education, BMI, smoking status, drinking status, dietary patterns, and symptom severity to build the model, it exhibited great positive net benefits.

Several limitations of the study should be considered. First, there may be recall bias in our study, which is the common issue in cross-sectional studies. The community-level recruitment approach in this report reduces but does not eliminate this source of bias. Second, our model has only been internally validated, and the model will need to be further externally validated in the future if possible. Third, cigarette smoking is known risk factor for ESCC. Due to non-availability of the data of detailed tobacco consumption between the cases and controls, there may exist residual confounding from smoking, thus reducing the OR of the dietary patterns and severity of symptom with the risk of the ESD and ESCC. In addition, due to lack of information on genetics, we have not investigate the genetic impact on esophageal squamous carcinoma development.

## Conclusions

In conclusion, LCA gives further insights into dietary patterns and symptoms research, allowing for the definition and estimation of the prevalence of different groups of subjects characterized by different dietary choices and symptoms, and comparing those groups in relation to important health outcomes. Individuals at high risk of ESCC might be strongly recommended a “Healthy pattern” in the future life. Additionally, more dietary nutrition interventions and health promotion would be improved for the precision prevention, which is imperative to reduce the incidence and mortality of ESCC.

## Supplementary Information

**Additional file 1: Supplementary Table 1**. Probabilities of consumption for selected food items by dietary patterns derived from LCA for normal control and esophagitis. **Supplementary Table 2**. Probabilities of consumption for selected food items by dietary patterns derived from LCA for normal control and LGIN. **Supplementary Table 3**. Probabilities of consumption for selected food items by dietary patterns derived from LCA for normal control and HGIN&ESCC. **Supplementary Table 4**. Probabilities for selected symptom items by severity of symptom derived from LCA for normal control and esophagitis. **Supplementary Table 5**. Probabilities for selected symptom items by severity of symptom derived from LCA for normal control and LGIN. **Supplementary Table 6**. Probabilities for selected symptom items by severity of symptom derived from LCA for normal control and HGIN&ESCC.

## Data Availability

The datasets used and/or analysed during the current study are available from the corresponding author on reasonable request.

## Abbreviations

ESCC: Esophageal squamous cell carcinoma
LCA: Latent class analysis
ORs: Odds ratio
CIs: Confidence intervals
EADC: Esophageal adenocarcinoma
EPL: Esophageal precancerous lesions
LGIN: Low-grade intraepithelial neoplasia
HGIN: High-grade intraepithelial neoplasia
PCA: Principal component analysis
FA: Factor analysis
ESSH: Esophageal squamous simple hyperplasia
ESD: Esophageal squamous dysplasia
AIC: Akaike information criterion
BIC: Bayesian information criterion
BMI: Body mass index
