# Supplementary material for: Dietary patterns and severity of symptom with the risk of esophageal squamous cell carcinoma and its histological precursor lesions in China: a multicenter cross-sectional latent class analysis
Source: BMC Cancer. 2022 Jan 21;22:95. doi: 10.1186/s12885-022-09206-y (PMC8783423; doi:10.1186/s12885-022-09206-y)
Supplement: Supplementary file 1 — Additional file 1: Supplementary Table 1. Probabilities of consumption for selected food items by dietary patterns derived from LCA for normal control and esophagitis. Supplementary Table 2. Probabilities of consumption for selected food items by dietary patterns derived from LCA for normal control and LGIN. Supplementary Table 3. Probabilities of consumption for selected food items by dietary patterns derived from LCA for normal control and HGIN&ESCC. Supplementary Table 4. Probabilities for selected symptom items by severity of symptom derived from LCA for normal control and esophagitis. Supplementary Table 5. Probabilities for selected symptom items by severity of symptom derived from LCA for normal control and LGIN. Supplementary Table 6. Probabilities for selected symptom items by severity of symptom derived from LCA for normal control and HGIN&ESCC. [file 12885_2022_9206_MOESM1_ESM.docx]

Supplementary Table 1 Probabilities of consumption for selected food items by dietary patterns derived from LCA for normal control and esophagitis

| Food items | | Prudent | Western | Lower consumers-  combination | Medium consumers-  combination | Higher consumers-  combination |
| --- | --- | --- | --- | --- | --- | --- |
| Livestock meat and  its products | everyday | 76.9% | 84.3% | 15.2% | 9.4% | 55.2% |
|  | 1-6days/week | 12.8% | 12.5% | 22.1% | 72.7% | 42.0% |
|  | <1day/week | 10.3% | 3.1% | 62.8% | 17.9% | 2.7% |
| Poultry meat | ≥1day/week | 7.0% | 7.2% | 1.5% | 3.5% | 89.1% |
|  | <1day/week | 93.0% | 92.8% | 98.5% | 96.5% | 10.9% |
| Seafood | ≥1day/week | 1.4% | 9.2% | 1.5% | 0.4% | 64.2% |
|  | <1day/week | 98.6% | 90.8% | 98.5% | 99.6% | 35.8% |
| Eggs and its products | everyday | 60.8% | 72.4% | 22.2% | 32.0% | 50.8% |
|  | 1-6days/week | 15.6% | 16.6% | 23.9% | 64.5% | 37.9% |
|  | <1day/week | 23.6% | 11.1% | 53.9% | 3.5% | 11.4% |
| Vegetables | everyday | 99.8% | 0.0% | 2.5% | 2.8% | 97.1% |
|  | 1-6days/week | 0.1% | 0.7% | 2.6% | 97.2% | 2.4% |
|  | <1day/week | 0.1% | 99.3% | 94.9% | 0.0% | 0.6% |
| Fruits | everyday | 41.8% | 18.7% | 3.1% | 2.9% | 32.2% |
|  | 1-6days/week | 21.3% | 42.0% | 12.1% | 59.6% | 53.9% |
|  | <1day/week | 36.8% | 39.3% | 84.7% | 37.5% | 13.9% |
| Bean products | ≥1day/week | 38.2% | 45.7% | 8.6% | 45.5% | 65.9% |
|  | <1day/week | 61.8% | 54.3% | 91.4% | 54.5% | 34.1% |
| Scallion, ginger and garlic | ≥1day/week | 93.9% | 72.7% | 36.9% | 59.8% | 84.3% |
|  | <1day/week | 6.1% | 27.3% | 63.1% | 40.2% | 15.7% |
| Pickles | ≥1day/week | 4.9% | 68.7% | 2.9% | 11.4% | 17.6% |
|  | <1day/week | 95.1% | 31.3% | 97.1% | 88.6% | 82.4% |
| Nut fruits | ≥1day/week | 17.2% | 33.9% | 2.0% | 4.9% | 24.0% |
|  | <1day/week | 82.8% | 66.1% | 98.0% | 95.1% | 76.0% |
| Cluster’s size | | 29.1% | 9.2% | 28.9% | 28.7% | 4.1% |

Supplementary Table 2 Probabilities of consumption for selected food items by dietary patterns derived from LCA for normal control and LGIN

| Food items | | Prudent | Western | Lower consumers-  combination | Medium consumers-  combination | Higher consumers-  combination |
| --- | --- | --- | --- | --- | --- | --- |
| Livestock meat and  its products | everyday | 77.3% | 85.1% | 15.9% | 10.7% | 58.1% |
|  | 1-6days/week | 12.5% | 11.9% | 22.2% | 70.8% | 39.7% |
|  | <1day/week | 10.2% | 3.0% | 61.9% | 18.5% | 2.2% |
| Poultry meat | ≥1day/week | 7.0% | 6.7% | 1.6% | 3.5% | 87.9% |
|  | <1day/week | 93.0% | 93.3% | 98.4% | 96.5% | 12.1% |
| Seafood | ≥1day/week | 1.4% | 8.7% | 1.4% | 0.4% | 61.9% |
|  | <1day/week | 98.6% | 91.3% | 98.6% | 99.6% | 38.1% |
| Eggs and its products | everyday | 60.3% | 73.3% | 23.4% | 33.4% | 51.6% |
|  | 1-6days/week | 15.4% | 16.2% | 22.1% | 62.7% | 36.6% |
|  | <1day/week | 24.3% | 10.6% | 54.5% | 3.9% | 11.8% |
| Vegetables | everyday | 99.8% | 0.0% | 2.6% | 2.6% | 97.5% |
|  | 1-6days/week | 0.1% | 0.6% | 2.6% | 97.4% | 2.6% |
|  | <1day/week | 0.1% | 99.4% | 94.9% | 0.0% | 0.4% |
| Fruits | everyday | 42.4% | 20.0% | 3.3% | 3.2% | 32.9% |
|  | 1-6days/week | 20.7% | 41.0% | 11.7% | 58.9% | 53.0% |
|  | <1day/week | 36.9% | 39.0% | 84.9% | 37.9% | 14.1% |
| Bean products | ≥1day/week | 38.5% | 46.1% | 6.5% | 43.3% | 67.0% |
|  | <1day/week | 61.5% | 53.9% | 93.5% | 56.7% | 33.0% |
| Scallion, ginger and garlic | ≥1day/week | 94.8% | 72.7% | 38.6% | 60.1% | 85.3% |
|  | <1day/week | 5.2% | 27.3% | 61.4% | 39.9% | 14.7% |
| Pickles | ≥1day/week | 4.2% | 65.8% | 3.1% | 11.5% | 18.7% |
|  | <1day/week | 95.8% | 34.2% | 96.9% | 88.5% | 81.3% |
| Nut fruits | ≥1day/week | 17.1% | 33.0% | 2.0% | 5.3% | 25.5% |
|  | <1day/week | 82.9% | 67.0% | 98.0% | 94.7% | 74.5% |
| Cluster’s size | | 29.5% | 10.0% | 27.4% | 29.0% | 4.2% |

Supplementary Table 3 Probabilities of consumption for selected food items by dietary patterns derived from LCA for normal control and HGIN&ESCC

| Food items | | Prudent | Western | Lower consumers-  combination | Medium consumers-  combination | Higher consumers-  combination |
| --- | --- | --- | --- | --- | --- | --- |
| Livestock meat and  its products | everyday | 77.2% | 84.5% | 16.3% | 10.4% | 59.1% |
|  | 1-6days/week | 12.7% | 12.2% | 22.1% | 71.6% | 38.6% |
|  | <1day/week | 10.1% | 3.3% | 61.6% | 18.0% | 2.4% |
| Poultry meat | ≥1day/week | 6.9% | 6.9% | 1.6% | 3.7% | 87.7% |
|  | <1day/week | 93.1% | 93.1% | 98.4% | 96.3% | 12.3% |
| Seafood | ≥1day/week | 1.4% | 8.8% | 1.3% | 0.4% | 60.9% |
|  | <1day/week | 98.6% | 91.2% | 98.7% | 99.6% | 39.1% |
| Eggs and its products | everyday | 59.9% | 72.9% | 23.6% | 33.9% | 52.0% |
|  | 1-6days/week | 15.7% | 16.1% | 22.3% | 62.6% | 36.0% |
|  | <1day/week | 24.3% | 11.0% | 54.2% | 3.5% | 12.0% |
| Vegetables | everyday | 99.8% | 0.0% | 2.6% | 2.6% | 97.5% |
|  | 1-6days/week | 0.1% | 0.7% | 2.5% | 97.4% | 2.1% |
|  | <1day/week | 0.1% | 99.3% | 94.9% | 0.0% | 0.4% |
| Fruits | everyday | 42.8% | 20.2% | 3.5% | 3.4% | 33.6% |
|  | 1-6days/week | 20.9% | 41.5% | 12.2% | 59.0% | 51.9% |
|  | <1day/week | 36.3% | 38.3% | 84.3% | 37.7% | 14.5% |
| Bean products | ≥1day/week | 38.7% | 45.9% | 6.7% | 43.6% | 67.5% |
|  | <1day/week | 61.3% | 54.1% | 93.3% | 56.4% | 32.5% |
| Scallion, ginger and garlic | ≥1day/week | 94.9% | 72.7% | 39.1% | 60.2% | 85.1% |
|  | <1day/week | 5.1% | 27.3% | 60.9% | 39.8% | 14.9% |
| Pickles | ≥1day/week | 4.2% | 66.2% | 3.2% | 11.0% | 18.2% |
|  | <1day/week | 95.8% | 33.8% | 96.8% | 89.0% | 81.8% |
| Nut fruits | ≥1day/week | 17.3% | 33.8% | 2.0% | 5.2% | 24.9% |
|  | <1day/week | 82.7% | 66.2% | 98.0% | 94.8% | 75.1% |
| Cluster’s size | | 29.6% | 9.8% | 27.4% | 28.9% | 4.2% |

Supplementary Table 4 Probabilities of consumption for selected symptom items by severity of symptom derived from LCA for normal control and esophagitis

| Symptom items | | Asymptomatic | Mild symptoms | Overt symptoms |
| --- | --- | --- | --- | --- |
| Number of lost teeth | 0 teeth | 48.9% | 10.8% | 32.9% |
|  | 1-3 teeth | 29.5% | 71.6% | 32.5% |
|  | more than 4 teeth | 21.7% | 17.6% | 34.6% |
| Whether gingival bleeding | Yes | 22.0% | 100.0% | 38.4% |
|  | No | 78.0% | 0.0% | 61.6% |
| Whether dysphagia | Yes | 0.2% | 0.1% | 6.5% |
|  | No | 99.8% | 99.9% | 93.5% |
| Whether bloating, heartburn, acid reflux | Yes | 1.6% | 0.0% | 45.3% |
|  | No | 98.4% | 100.0% | 54.7% |
| Whether nausea, vomiting and belching | Yes | 0.6% | 1.1% | 35.4% |
|  | No | 99.4% | 98.9% | 64.6% |
| Whether epigastric pain | Yes | 0.8% | 0.2% | 19.4% |
|  | No | 99.2% | 99.8% | 80.6% |
| Cluster’s size | | 90.7% | 6.0% | 3.4% |

Supplementary Table 5 Probabilities of consumption for selected symptom items by severity of symptom derived from LCA for normal control and LGIN

| Symptom items | | Asymptomatic | Mild symptoms | Overt symptoms |
| --- | --- | --- | --- | --- |
| Number of lost teeth | 0 teeth | 49.1% | 40.7% | 32.0% |
|  | 1-3 teeth | 29.6% | 39.1% | 32.6% |
|  | more than 4 teeth | 21.3% | 20.3% | 35.4% |
| Whether gingival bleeding | Yes | 0.0% | 100.0% | 35.5% |
|  | No | 100.0% | 0.0% | 64.5% |
| Whether dysphagia | Yes | 0.2% | 0.1% | 6.8% |
|  | No | 99.8% | 99.9% | 93.2% |
| Whether bloating, heartburn, acid reflux | Yes | 1.6% | 1.4% | 48.3% |
|  | No | 98.4% | 98.6% | 51.7% |
| Whether nausea, vomiting and belching | Yes | 0.6% | 1.0% | 36.0% |
|  | No | 99.4% | 99.0% | 64.0% |
| Whether epigastric pain | Yes | 0.8% | 0.9% | 20.1% |
|  | No | 99.2% | 99.1% | 79.9% |
| Cluster’s size | | 72.0% | 26.5% | 1.5% |

Supplementary Table 6 Probabilities of consumption for selected symptom items by severity of symptom derived from LCA for normal control and HGIN&ESCC

| Symptom items | | Asymptomatic | Mild symptoms | Overt symptoms |
| --- | --- | --- | --- | --- |
| Number of lost teeth | 0 teeth | 49.5% | 0.0% | 31.5% |
|  | 1-3 teeth | 29.3% | 86.9% | 33.5% |
|  | more than 4 teeth | 21.1% | 13.1% | 35.0% |
| Whether gingival bleeding | Yes | 23.2% | 100.0% | 38.1% |
|  | No | 76.8% | 0.0% | 61.9% |
| Whether dysphagia | Yes | 0.2% | 0.2% | 6.8% |
|  | No | 99.8% | 99.8% | 93.2% |
| Whether bloating, heartburn, acid reflux | Yes | 1.6% | 0.0% | 45.2% |
|  | No | 98.4% | 100.0% | 54.8% |
| Whether nausea, vomiting and belching | Yes | 0.6% | 0.9% | 35.4% |
|  | No | 99.4% | 99.1% | 64.6% |
| Whether epigastric pain | Yes | 0.8% | 0.3% | 19.6% |
|  | No | 99.2% | 99.7% | 80.4% |
| Cluster’s size | | 92.1% | 4.3% | 3.6% |
